# Localization of the CyanoP binding site on photosystem II by surface plasmon resonance spectroscopy

**DOI:** 10.3389/fpls.2014.00595

**Published:** 2014-11-05

**Authors:** Kai U. Cormann, Maik Bartsch, Matthias Rögner, Marc M. Nowaczyk

**Affiliations:** Plant Biochemistry, Ruhr University BochumBochum, Germany

**Keywords:** photosynthesis, photosystem II, CyanoP, PsbP, surface plasmon resonance (SPR), assembly factor, photoactivation

## Abstract

Photosystem II (PSII), a large multi subunit membrane protein complex localized in the thylakoid membrane of cyanobacteria and chloroplasts, is the only known enzyme that catalyzes the light-driven oxidation of water. In addition to the membrane intrinsic part of PSII, efficient oxygen evolution requires soluble protein subunits at its luminal interface. In contrast to the detailed crystal structure of the active cyanobacterial complex the characterization of intermediate PSII species related to its assembly and repair is hampered by their instability or low abundance. As most structural variations of the corresponding PSII species are based on a different set of protein factors bound to the luminal interface of the complex we developed a system for interaction analysis between PSII and its soluble interaction partners based on surface plasmon resonance (SPR) spectroscopy. The assay was validated by the correct localization of the extrinsic PSII proteins PsbO, PsbV, and PsbU on the luminal PSII surface and used to determine the unknown binding position of CyanoP, the cyanobacterial homolog of higher plant PsbP. The CyanoP binding site was clearly localized in the center of PSII at a position, which is occupied by the PsbO subunit in mature PSII complexes. Consistently, we demonstrate selective binding of CyanoP to an inactive PSII assembly intermediate that lacks the extrinsic subunits PsbO, PsbV, and PsbU. These findings suggest, that CyanoP functions in the dynamic lifecycle of PSII, possibly in the association of CP47 and CP43 or in photoactivation of the oxygen-evolving complex.

## INTRODUCTION

Photosystem II (PSII) catalyzes one of nature’s key reactions: the light-driven oxidation of water. Remarkable advances in X-ray crystallography of cyanobacterial PSII improved the structural models of this multisubunit pigment-protein complex to a resolution of 1.9 Å (Umena et al., 2011). In total 19 protein subunits and multiple cofactors like chlorophylls, carotenoids, lipids, metal ions, and the oxygen-evolving complex (OEC), the catalytic center of the water-splitting reaction, have been elucidated in this recent model. Out of the 19 subunits, 16 proteins are embedded in the thylakoid membrane, whereas the three extrinsic proteins PsbO, PsbV, and PsbU are solvent accessible, protruding out into the thylakoid lumen. Notably, the crystal structure depicts only one out of various PSII complexes. Those which represent assembly and repair intermediates of the PSII lifecycle (Komenda et al., 2012), are known to contain a makeup of extrinsic factors different from that of the fully assembled complex (Nowaczyk et al., 2006; Rengstl et al., 2011). For instance, Psb27, a lipoprotein associated with CP43 (Liu et al., 2011), is only transiently bound to the PSII complex; it appears to have a role in the assembly and repair process of PSII after photodamage (Nowaczyk et al., 2006; Grasse et al., 2011). Another example includes CtpA, a sequence specific protease, which cleaves a C-terminal extension from the D1-precursor subunit in the early phase of PSII biogenesis (Anbudurai et al., 1994), a prerequisite for the assembly of the OEC.

In addition, the composition of the extrinsic subunits has changed over the course of evolution of photosynthetic organisms (Nagarajan and Burnap, 2012). In contrast to PsbO, which is common for all photoautotrophs, the presence of PsbV and PsbU is restricted to cyanobacteria (Shen et al., 1992), red algae (Enami et al., 1995), and diatoms (Nagao et al., 2010b), while PSII of green algae (Suzuki et al., 2003) and higher plants (Murata et al., 1984) contains stoichiometric amounts of PsbP and PsbQ. Interestingly, cyanobacterial homologs of these subunits (CyanoP and CyanoQ, respectively) have been found in PSII preparations from *Synechocystis* sp. PCC 6803 (Thornton et al., 2004), and CyanoQ was shown to be specific for highly active PSII complexes (Roose et al., 2007). However, both proteins are missing in PSII purified from *Thermosynechococcus vulcanus*, which has been crystallized (Umena et al., 2011).

The impact of these various compositions on the PSII structure is difficult to elucidate due to their low abundance, transient nature or instability. In particular, the structural characterization of CyanoP PSII complexes is hampered by their low abundance in cyanobacterial PSII preparations (Thornton et al., 2004; Ishikawa et al., 2005). This may be due to loss during purification (Ishikawa et al., 2005) or the small amount of CyanoP containing PSII species (Thornton et al., 2004). In contrast to the well characterized function of higher plant PsbP in maintaining PSII activity (Ifuku et al., 2005; Yi et al., 2007; Ido et al., 2012) and regulating the binding of PsbQ (Kakiuchi et al., 2012), the physiological role of CyanoP is still unknown. This is surprising as CyanoP represents the phylogenetic origin of the whole PsbP superfamily (Sato, 2010) and the structures of CyanoP (Michoux et al., 2010; Jackson et al., 2012) and PsbP (Ifuku et al., 2004) share a high degree of similarity. Here we introduce an in vitro assay based on surface plasmon resonance (SPR) spectroscopy for the localization of transiently bound proteins on large (membrane) protein complexes. While PsbP is a structural component of the active PSII complex in green algae and plants, our results indicate a role of CyanoP in the dynamic PSII lifecycle, presumably in the association of CP47 and CP43 or in photoactivation of the OEC.

## MATERIALS AND METHODS

### POLYMERASE CHAIN REACTION (PCR) AND MOLECULAR CLONING

Gene sequences from *Thermosynechococcus elongatus* coding for CyanoP, CyanoQ, PsbO, PsbU, PsbV, and Psb27 without signal peptide and the luminal domains of precursor D1 (pD1), mature D1 (mD1), D1 a-loop (D1a), D1 peptide (D1pep), D2, CP43, CP47, and PsbE were amplified by PCR using oligonucleotides summarized in Table S1. Details of the cloning procedure for the generation of expression templates for the immunity protein 7 (Im7) fusion proteins, CyanoP, PsbO, and PsbV are given in the supplementary material.

### CELL-FREE PROTEIN EXPRESSION

Cell-free expression of the Im7 tagged luminal domains was done according to the manufacturer’s instructions using the RTS100system (5Prime). Briefly, 500 ng of expression plasmid were added to the reaction mixture. The expression reaction was performed by incubation for 6 h at 30°C under slight shaking (100 rpm). Aliquots were stored at -80°C.

### HETEROLOGOUS PROTEIN OVEREXPRESSION AND PURIFICATION

Heterologous overexpression of CyanoP, PsbO, and Im7 fusion proteins of PsbO, PsbU, Psb27, CyanoP, and CyanoQ was performed with *Escherichia coli* Overexpress C43 cells (Lucigen) and the corresponding expression vector derived from pIVEX2.4d (5Prime). A more detailed description of the expression and purification procedure is given in the supplementary material. Expression of PsbV was based on a previously published protocol (Andrews et al., 2005). Details on the purification of the strep-tagged protein are given in the supplementary material. Expression and purification of DNase E7 was carried out according to Hosse et al. (2009).

### MASS SPECTROMETRY

Sample preparation and mass spectrometric analysis for identification of the Im7 fusion proteins was done according to Nowaczyk et al. (2011). An *E. coli* K12 protein database supplemented with the sequences for the Im7 fusion proteins or a *T. elongatus* protein database was used in this approach.

### SPR EXPERIMENTS

All SPR measurements were performed with a Biacore3000 instrument using CM5 sensor chips (both GE Healthcare). Preparation of DNase E7 coated surfaces was done according to Hosse et al. (2009). Additionally, the surface was conditioned with two consecutive 1 min injections of Gentle Elution Buffer (Thermo Scientific) at a flow rate of 60 μl/min. Details of the on-chip purification of Im7 fusion proteins and the SPR interaction analysis are given in the supplementary material.

### PREPARATION OF PSII AND RECONSTITUTION EXPERIMENTS

Preparation of PSII complexes from *T. elongatus* (wildtype) was done according to Kuhl et al. (2000), Nowaczyk et al. (2006). For reconstitution of PSII with recombinant CyanoP inactive monomeric PSII and highly active dimeric PSII (Nowaczyk et al., 2006; both at 0.1 mg/ml chlorophyll) were incubated with a twofold molar excess of CyanoP (15 min; 4°C) in buffer A (20 mM MES, 10 mM MgCl_2_, 10 mM CaCl_2_, 0.03 % (w/v) n-Dodecyl β-D-maltoside, pH 6.5). Unbound CyanoP was removed by three washing steps with 250 μl buffer A using centrifugal filter devices (Microcon YM100, Millipore; 6.000 g, 10 min, 4°C). Reconstitution experiments were performed as two independent biological replicates using different PSII preparations.

## RESULTS

### THE LUMINAL INTERFACE OF PSII CAN BE MIMICKED BY RECOMBINANT PROTEIN DOMAINS

**FIGURE 1:**
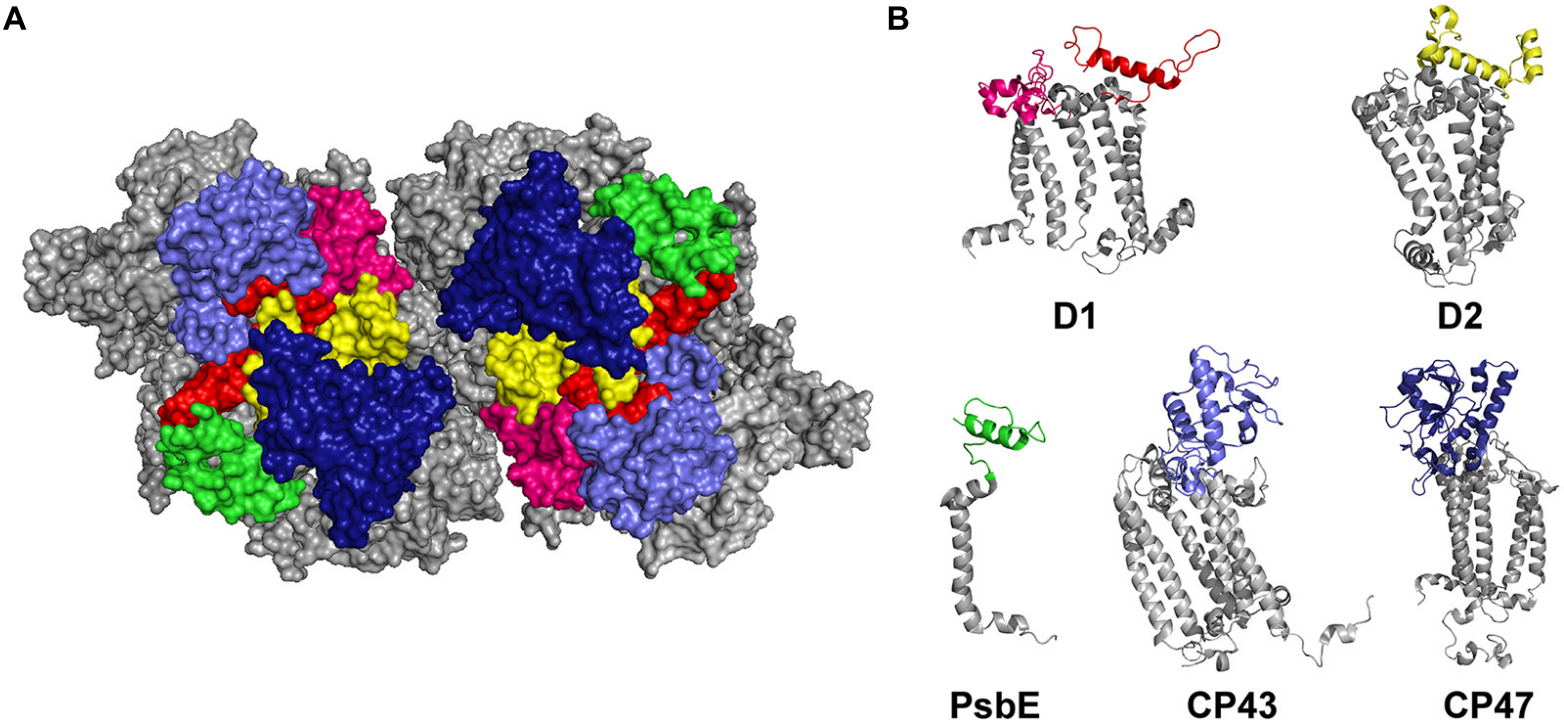
**The luminal interaction interface of PSII. (A)** Top view of the luminal side of dimeric PSII without extrinsic subunits (Umena et al., 2011). The e-loops of CP43 (light blue), CP47 (dark blue), the a-loop of D1 (pink) and the C-terminal domains of D1 (red), D2 (yellow), and PsbE (green) represent the major interaction sites for soluble subunits. **(B)** Cartoon showing the individual PSII subunits of which the colored domains can be expressed as recombinant proteins to be used for biomolecular interaction analysis with extrinsic PSII subunits or assembly factors.

**FIGURE 2:**
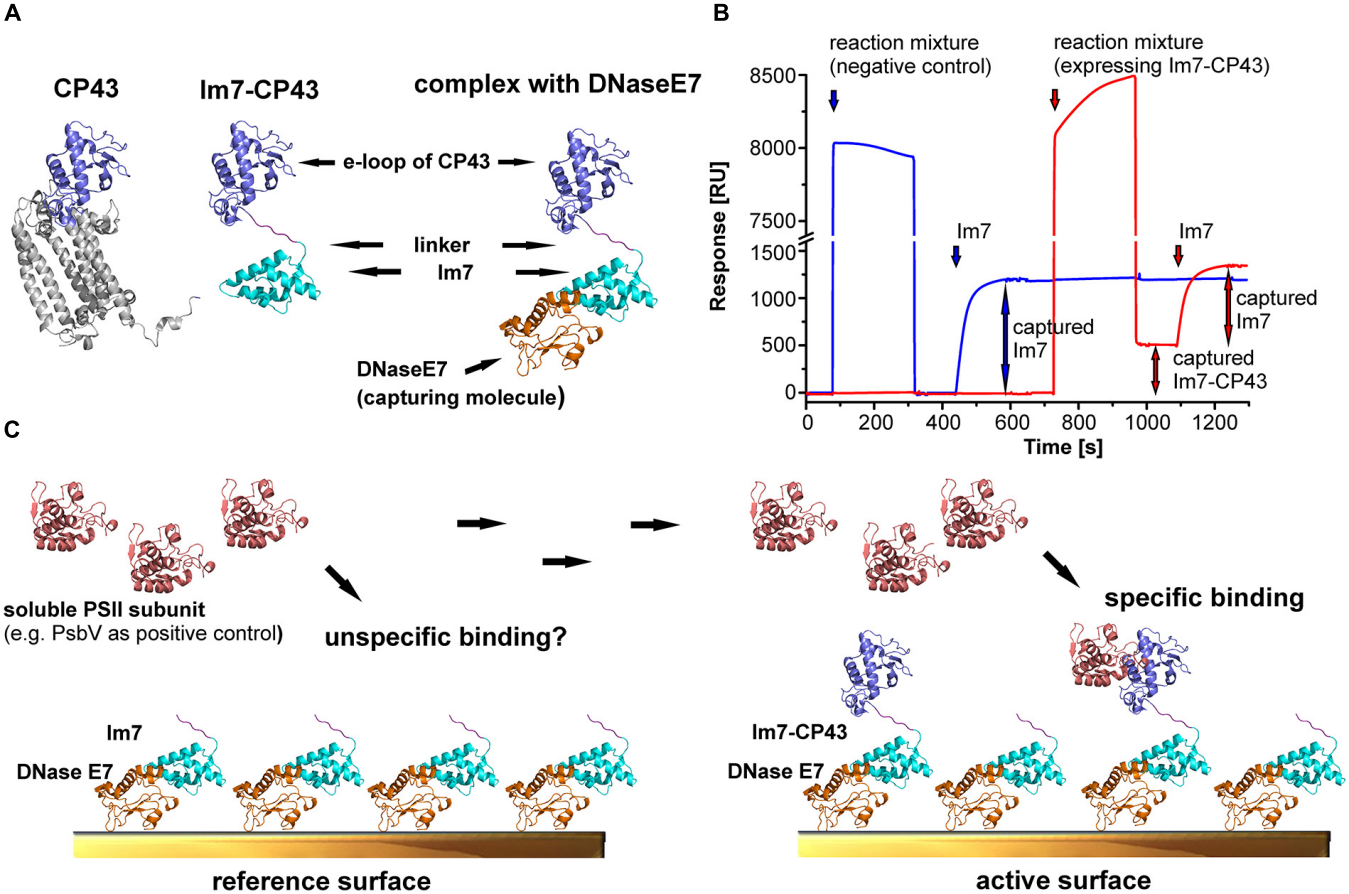
**Experimental approach for mapping the binding sites of extrinsic subunits and assembly factors of PSII.** The structural models are based on the crystal structures of PSII (Umena et al., 2011; pdb code: 3ARC) and the complex between DNase E7 and Im7 (Ko et al., 1999; pdb code: 7CEI). **(A)** Structural models for CP43, the e-loop of CP43 tagged with Im7 and the Im7-CP43 fusion protein bound to DNase E7. The Im7-tag (cyan) and DNase E7 (orange) are located in a position occupied by the transmembrane helices of native CP43 (gray), and thus neither the tag nor DNase E7 interfere with the binding of soluble interaction partners to the e-loop (light blue). **(B)** Preparation of active (red) and reference surface (blue) for surface plasmon resonance (SPR) interaction analysis. Unspecific binding of the reaction mixture to the surface is checked by injecting a 1000-fold dilution over the reference cell, upon which the signal returned to the baseline level. This indicates the absence of unspecific binding. In contrast, injection of a reaction mixture (identical dilution) expressing Im7-CP43 results in stable immobilization of 510 RU of fusion protein. Accordingly, the purity of the immobilized PSII domain on the SPR surface is close to 100%. Finally, both surfaces were saturated with purified Im7 (200 nM) to achieve maximal comparability between reference and active surface; division of the y-axis (high bulk signal caused by the high ionic strength of the immobilization buffer) should be considered. **(C)** Schematic structures of the surfaces prepared in **(B)**, which were used as positive control for SPR interaction analysis between Im7-CP43 [colors according to **(B)**] and PsbV (salmon). DNase E7 is covalently bound to the sensor surface, allowing stable immobilization of Im7 and Im7-tagged proteins. Unspecific binding is checked by injection of PsbV on a reference surface with immobilized Im7, whereas the sum of specific and unspecific binding is monitored on an active surface containing the Im7-tagged domain in the required amount. The reference-subtracted binding responses for interaction analysis between PSII domains and PsbV, PsbO or CyanoP are shown in **Figures 3–5**.

In the thylakoid lumen, most of the binding interface for the extrinsic subunits of PSII and the assembly factors is provided by the solvent exposed e-loops of CP43 and CP47, by the D1 a-loop, as well as by the C-termini of D1, D2, and PsbE (**Figure 1A**; **Table 1**). Hence, we decided to express these domains from *T. elongatus* (**Figure 1B**) as recombinant proteins and immobilize them on an SPR sensor surface to determine their affinity for their putative interaction partners. This approach is based on a previously reported capture system (Hosse et al., 2009) utilizing the tight interaction between the nuclease domain of colicinE7 (DNase E7) and its inhibitor, Im7. The latter was fused via a hydrophilic and flexible linker sequence (GGSG) to the N-terminus of the CP43/CP47 e-loops, D1a and the C-terminal domains of pD1, mD1, D1pep, D2, and PsbE (**Figure 2A**) allowing stable and selective immobilization of the fusion construct on DNase E7 coated surfaces (Hosse et al., 2009). Moreover, the structure of DNase E7 in complex with Im7 (Ko et al., 1999) suggests that the immobilized PSII domain is completely accessible for soluble proteins (**Figure 2A**). To ensure that the domains adopt a conformation which allows specific binding of their interaction partners, an extrinsic PSII subunit with known binding position (e.g., PsbV, PsbO) can be used as positive or negative control. This subunit is first injected over a reference surface coated with DNase E7 in complex with Im7 in order to check for unspecific binding to the sensor surface (**Figures 2B,C**). Subsequently, the same sample is exposed to an analogous surface with either an interacting (positive control) or non-interacting (negative control) Im7-PSII fusion protein. As shown in the following paragraphs this carefully referenced setup enables the precise detection of specific protein-protein interactions even if the corresponding binding affinities are in the intermediate micromolar range.

**Table 1:** Nomenclature of different D1 domains.

Abbreviation	Domain	Residues (PsbA1)
mD1	C-terminal D1 domain in its processed form	N295 – A344
pD1	C-terminal D1 domain with precursor peptide	N295 – G360
D1a	D1 a-loop	A54 – Q113
D1pep	C-terminal peptide of processed D1	H332 – A344

### Im7-FUSION PROTEINS OF THE LUMINAL PSII DOMAINS CAN BE PURIFIED ON THE SPR SENSOR SURFACE

The luminal PSII domains fused to Im7 were expressed in a cell-free system (RTS100, 5Prime) in order to avoid degradation of the truncated constructs, which was confirmed by SDS-PAGE and MS analysis (Figures S1 and S2). As cell-free expression yielded only a low amount of recombinant PSII domains, a sensor surface with immobilized DNase E7 (approximately 3500 response units, RU) was used as affinity matrix for the Im7-tagged constructs. The remarkable affinity and specificity of the Im7-E7 system enables on-chip purification and immobilization in one single step (**Figure 2B**) as confirmed by a control implemented in every experiment: the fact that an injection of a reaction mixture without template DNA over the reference surface did not affect the baseline level excludes unspecific binding to the sensor surface. In contrast, injection of a sample expressing the Im7-CP43 fusion protein increased the baseline on the active surface of the second flow cell by 510 RU. As the composition of both cell-free expression mixtures is identical – besides the presence of Im7-CP43 – this indicates a close to 100% purity of the Im7-tagged protein on the active surface. Also, in order to ensure maximal comparability between reference and active cell, both surfaces were saturated with purified Im7. Surface preparations with the other Im7-PSII fusion proteins showed identical purity and varied only in the amount of captured protein on the active surface due to differences in expression efficiency and molecular mass of the fused domains.

### RECOMBINANT PSII DOMAINS ARE SUITABLE FOR IDENTIFICATION OF THE MAJOR BINDING INTERFACES OF EXTRINSIC PSII SUBUNITS

**FIGURE 3:**
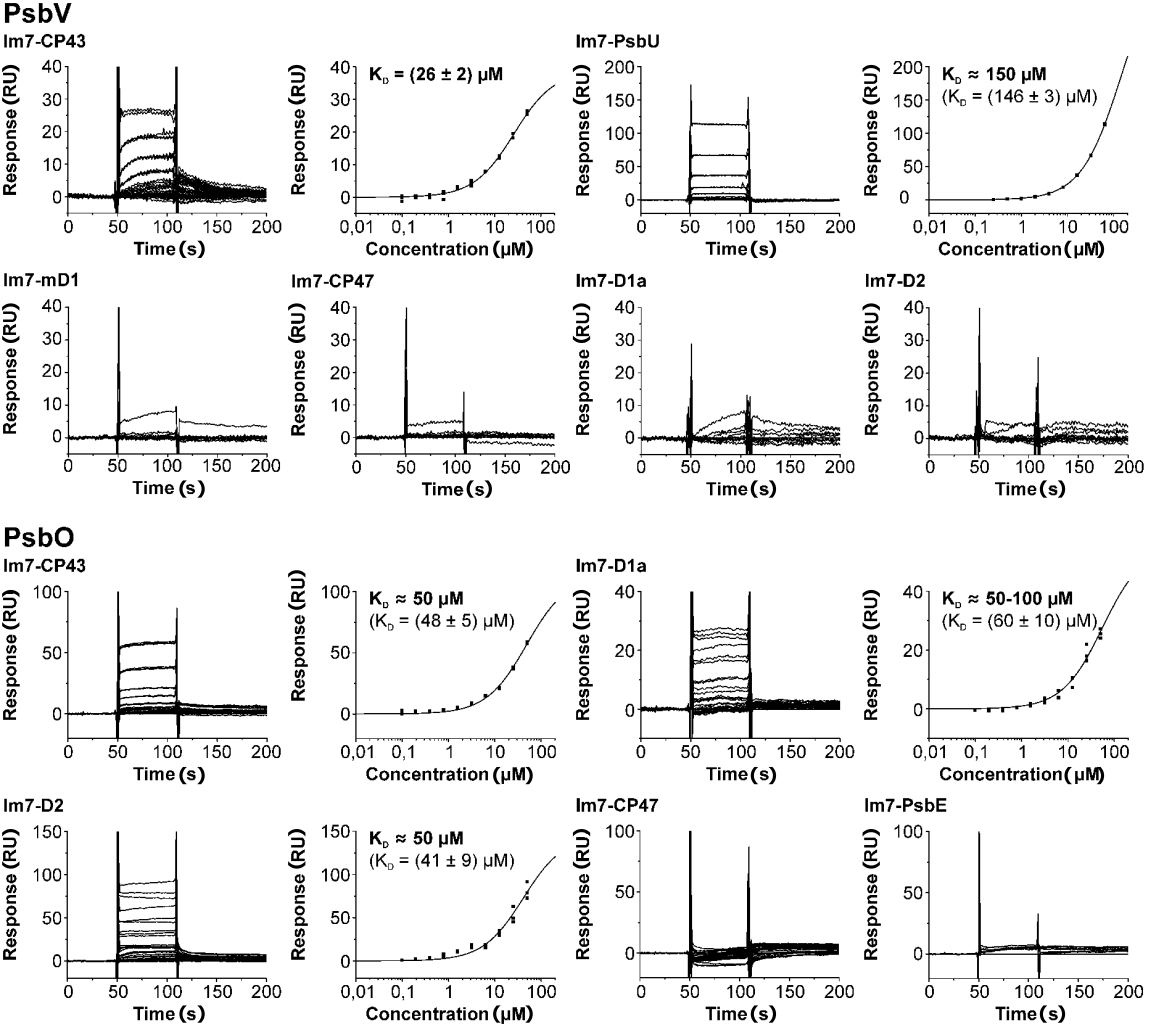
**Positive and negative controls for mapping the binding sites of soluble proteins at PSII by SPR interaction analysis.** Experiments were carried out with the known interaction partners of PsbV and PsbO derived from the PSII crystal structure (Umena et al., 2011; PsbV: CP43, mD1 and PsbU; PsbO: CP43, D1a, D2, and CP47). With two exceptions (PsbV and mD1, PsbO and CP47) triplicate sensograms for each analyte concentration reveal reproducible, concentration dependent steady state binding responses (left) that can be fitted to a one site binding isotherm (right, *K*_D_ given with SEs). Although each fit is in excellent agreement with the experimental data, most *K*_D_ values can only be considered as an approximation, as the highest analyte concentration is smaller than the calculated *K*_D_ (*K*_D_ values determined by the evaluation software are given in parenthese for these experiments). In the experiment with PsbV and CP43 one curve was omitted due to an instrumental error (Figure S3). Additionally, negative controls with non-interacting PSII domains were performed (PsbO and PsbE, PsbV and CP47, D1a or mD1, respectively). As expected no or negligible binding was observed.

The feasibility of our SPR based approach for mapping of protein binding sites on large (membrane) protein complexes was verified by analysis of the well-known binding position of PsbV and PsbO at the luminal surface of PSII. The PSII crystal structure (Umena et al., 2011) shows a major contact of PsbV with the CP43 e-loop and minor interactions with the C-terminus of D1 and the PsbU subunit. Indeed, a concentration dependent and reproducible binding to immobilized Im7-CP43 at PsbV concentrations ≥ 1 μM was observed (**Figure 3**). A plot of the steady state responses against the corresponding PsbV concentrations was fitted accurately to a one site binding isotherm yielding an affinity constant of 26 ± 2 μM. Although the reference surface can be used as intrinsic negative control for unspecific binding, the binding of PsbV to the e-loop of CP47, D1a and D2 was used as an additional control, yielding no binding except for a weak signal at 50 μM PsbV. In contrast to CP43, binding of PsbV to mD1 is similar to the unspecific interactions with the e-loop of CP47 and D1a, suggesting a weak contribution of D1 to the association of PsbV. The binding responses of PsbU, the third subunit with contact interfaces to PsbV (Umena et al., 2011), can be fitted to a one site binding model, but the calculated dissociation constant of 146 μM is > twofold higher than the highest concentration used and thus represents a rough estimate of the exact value (*K*_D_ ≈ 150 μM). This intermediate affinity is in agreement with data showing an enhancement of PsbU-binding to PSII by PsbV, albeit PsbO is additionally required for a complete association (Shen and Inoue, 1993). PsbO shows main contacts to CP43, D1a, D2, and CP47 according to the PSII crystal structure (Umena et al., 2011). The results of the SPR analysis were again in agreement with the binding position of PsbO derived from the PSII crystal structure. The PsbO subunit shows a concentration dependent and reproducible binding to immobilized Im7-CP43 (*K*_D_ ≈ 50 μM), Im7-D1a (*K*_D_ ≈ 50–100 μM), and Im7-D2 (*K*_D_ ≈ 50 μM) based on precisely fitted data (**Figure 3**) but not to Im7-CP47 and Im7-PsbE. The latter result of the control experiment is reasonable, as there are no contacts between PsbE and PsbO predicted from the PSII crystal structure (Umena et al., 2011). The undetectable binding of PsbO to Im7-CP47 might be explained by the small binding area between both proteins. In summary, the successful identification of PSII subunits with significant contribution to the PsbV and PsbO association shows the potential of this SPR based approach, although minor interaction interfaces – for instance PsbV with D1 and PsbO with CP47 – might be missed in the concentration range suitable for biomolecular interaction analysis.

### ASSOCIATION OF CyanoP WITH PSII IS DRIVEN BY ITS INTERACTION WITH THE C-TERMINAL DOMAIN OF D2 AND THE D1 a-LOOP

**FIGURE 4:**
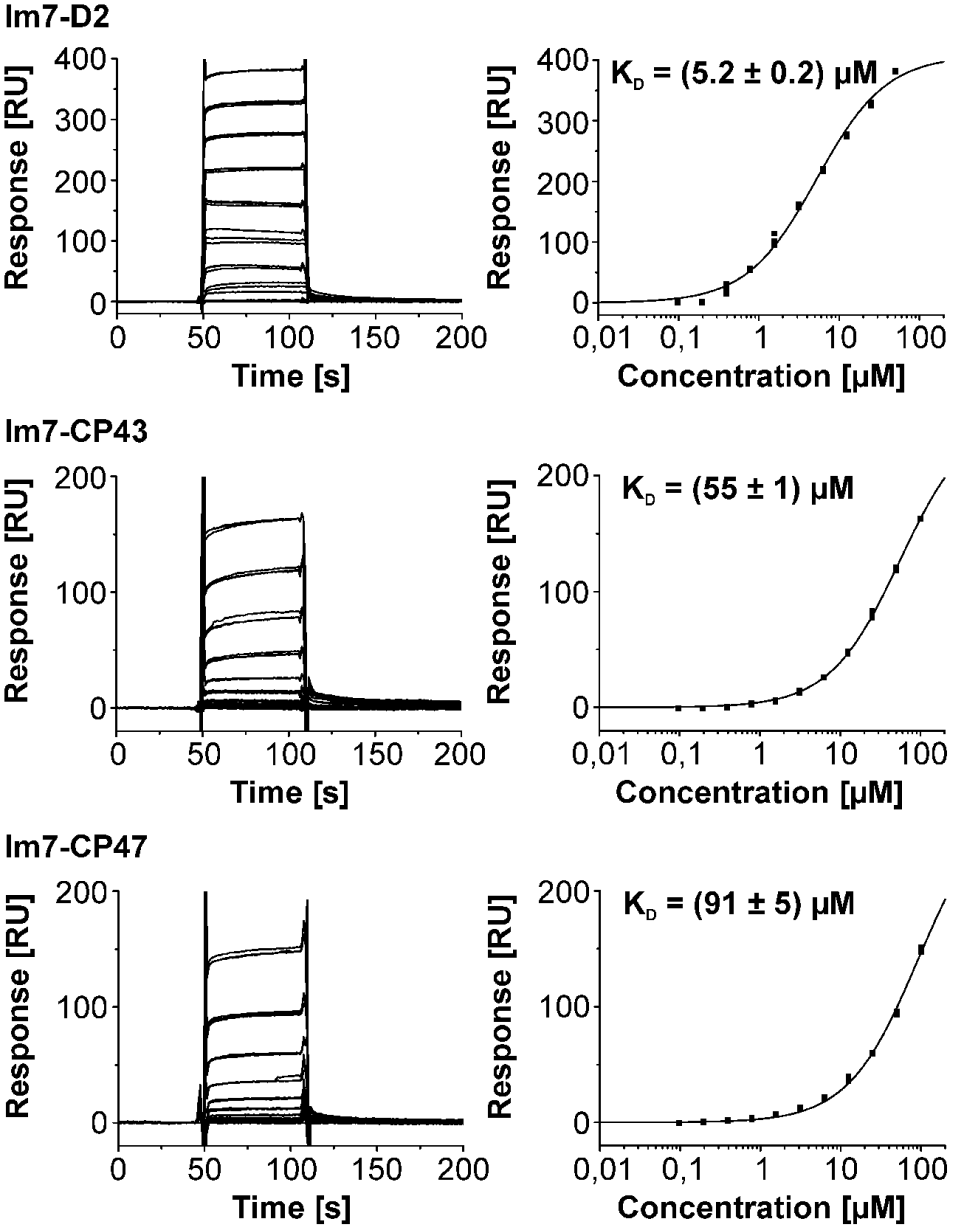
**Triplicate binding responses (left) and fits of steady state responses to a one site binding isotherm (right) of CyanoP interacting with Im7-D2, Im7-CP43, and Im7-CP47.** *K*_D_ values are given with SE. Each fit is in excellent agreement with the experimental responses as judged from the highest relative SE of 5.5% in the Im7-CP47 data set. Clearly, the affinity for the D2 domain exceeds all the other PSII subunits. Interaction analysis between CyanoP and different domains of D1 is shown in **Figure 5**.

**FIGURE 5:**
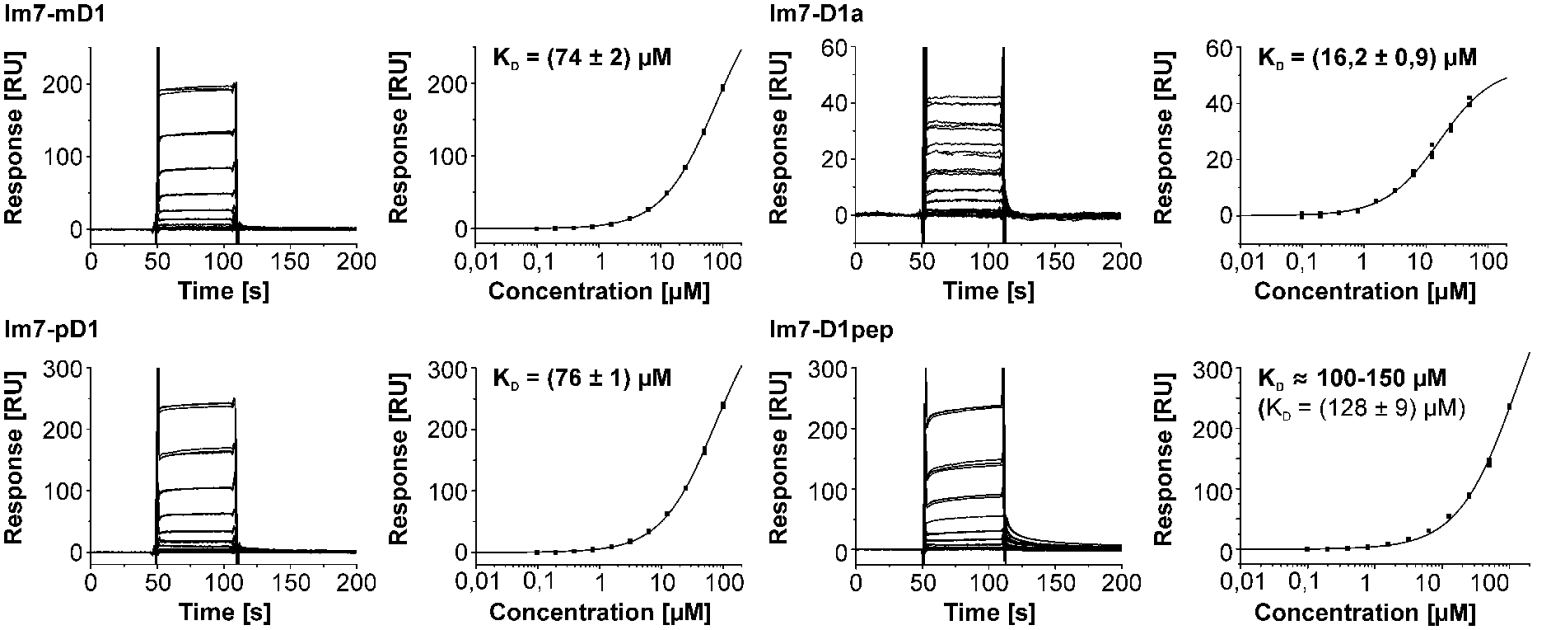
**Surface plasmon resonance interaction analysis between CyanoP and Im7 fusion proteins of pD1, mD1, D1a, and D1pep (peptide consisting of the 13 C-terminal residues of mature D1).** *K*_D_ values are given with SE. In the experiment with D1pep two curves were omitted due to an instrumental error (Figure S3). The affinity for D1a is the second highest measured for CyanoP. Moreover, the dissociation constants for Im7-mD1 and Im7-pD1 are – within range of error – identical.

**FIGURE 6:**
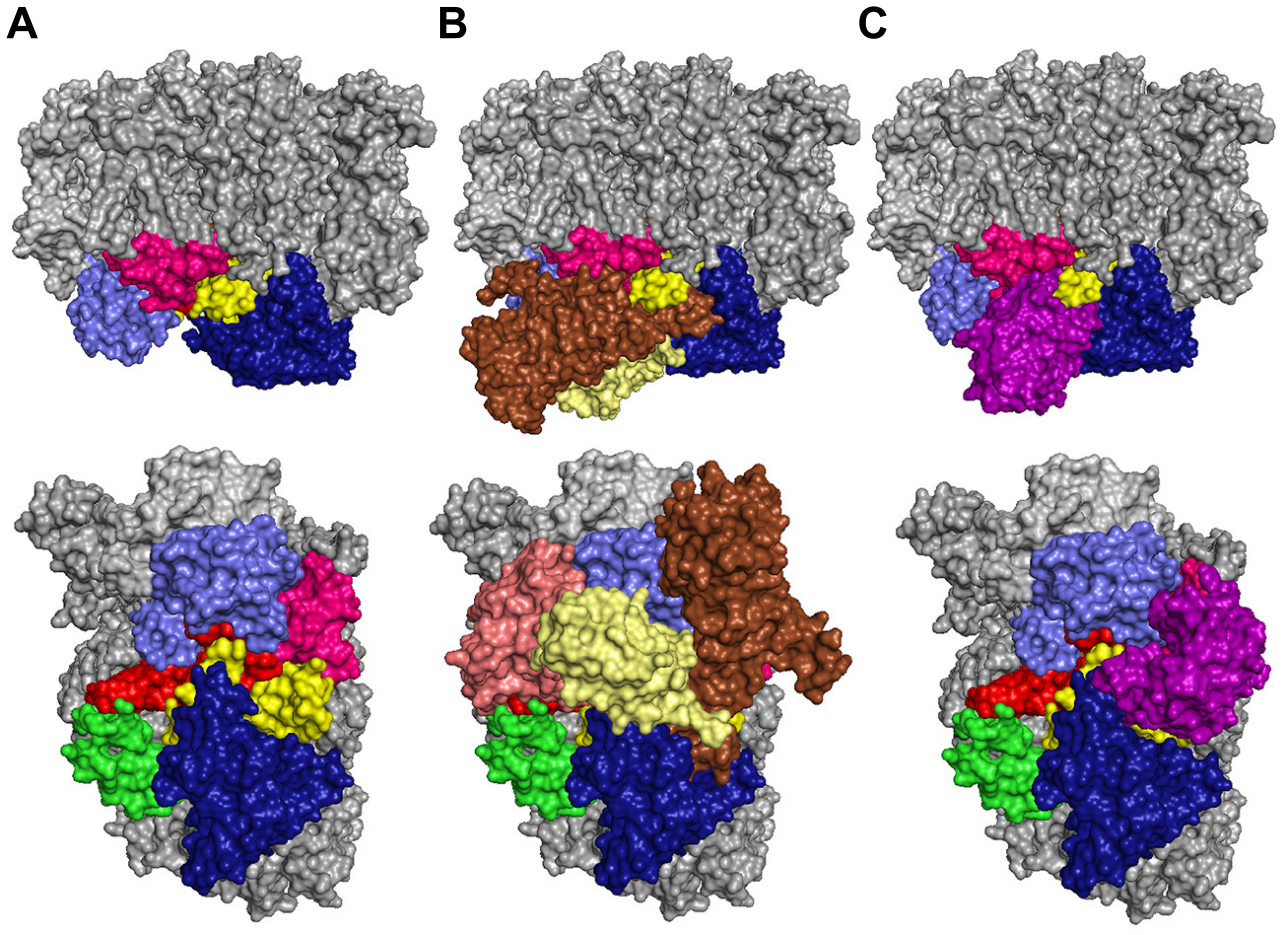
**Localization of CyanoP on PSII in side view (top) and top view (bottom).** The model is based on the respective crystal structures (Michoux et al., 2010; Umena et al., 2011; pdb codes: PSII: 3ARC; CyanoP: 2XB3). **(A)** The PSII structure without extrinsic subunits suggests a location of CyanoP in the center to enable simultaneous contacts to the luminal domains of D1 (red), D1a (pink) D2 (yellow), CP43 (light blue) and CP47 (dark blue). The C-terminus of PsbE is shown in green. **(B)** The extrinsic subunits PsbO (brown), PsbV (salmon) and PsbU (pale yellow) hide most of the interaction sites in the center of the complex. **(C)** Positioning of CyanoP (violet) at the binding site of PsbO is the only possible model: taking into consideration that the D1 C-terminus can adopt a different conformation in premature PSII complexes lacking the oxygen-evolving complex (OEC; **Figure 8**) it allows contact to all luminal domains which showed significant binding of CyanoP.

CyanoP was probed for interaction with various luminal PSII domains (CP43, CP47, mD1, D1pep, pD1, D1a, D2, and PsbE) and other putative binding partners (CyanoQ, PsbO, PsbV, PsbU, Psb27) by SPR (**Figures 4 and 5**; Figure S4). Besides PsbE (Figure S4), all luminal domains showed clear and reproducible binding responses, as judged by the overlay of triplicate sensograms for each concentration (**Figures 4 and 5**). The equilibrium responses are in excellent agreement with the fit to a one site binding isotherm yielding SEs of 5.5% of the dissociation constant. The highest affinities were measured between CyanoP and the C-terminal domain of D2 (5.2 ± 0.2 μM) and D1a (16.2 ± 0.9 μM) – both parts are located in the center of the complex (**Figure 6A**). A considerably lower affinity was determined for the flanking e-loops of CP43 (55 ± 1 μM) and CP47 (91 ± 5 μM), for pD1 (76 ± 1 μM), mD1 (74 ± 2 μM), and D1pep (*K*_D_ ≈ 100–150 μM). Within the range of error, these values indicate that the C-terminal extension of pD1 neither stabilizes nor prevents the association of CyanoP. Based on reconstitution experiments and cross-linking data, PsbP was proposed to interact with both PsbO and PsbQ in higher plants and green algae (Bricker and Frankel, 2003; Nagao et al., 2010a). Accordingly, we investigated interactions of CyanoP with the extrinsic cyanobacterial PSII subunits CyanoQ, PsbO, PsbV, PsbU, and Psb27, a factor involved in assembly and disassembly of PSII (Nowaczyk et al., 2006; Grasse et al., 2011). Although protein concentrations of up to 100 μM were used, no interaction was observed in these experiments (Figure S4). In conclusion, the CyanoP binding site was localized at the position of PsbO on the luminal PSII surface (**Figures 6B,C**).

### CyanoP SELECTIVELY INTERACTS WITH A PSII ASSEMBLY INTERMEDIATE

**FIGURE 7:**
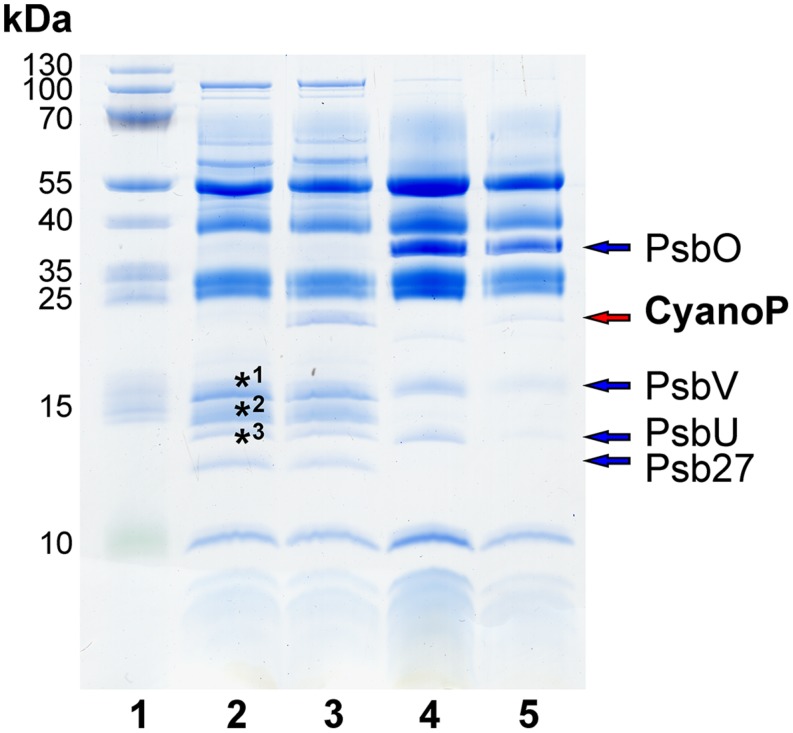
**Reconstitution of inactive monomeric and active dimeric PSII with CyanoP.** CyanoP is bound in nearly stoichiometric amounts to the monomer, whereas binding to the dimeric complex is negligible. Labeled bands were identified as Psb28 (*3) and ApcA, ApcB, CpcA, CpcB, (*1, *2) by mass spectrometry (Table S2). (lane 1: molecular weight marker; lane 2 and 3: PSII monomer before and after reconstitution with CyanoP; lane 4 and 5: PSII dimer before and after reconstitution with CyanoP).

For further analysis of the interaction between PSII and CyanoP we tested binding of recombinant CyanoP to highly active dimeric and inactive monomeric PSII from *T. elongatus*. In contrast to the dimer, which represents the main population of PSII and contains the extrinsic PsbO, PsbV, and PsbU subunits, the inactive monomer is associated to PSII assembly (Nowaczyk et al., 2006; Nowaczyk et al., 2012) and contains only Psb27 as extrinsic protein factor, which is associated to CP43 in close proximity to the binding site of PsbV (Liu et al., 2011). **Figure 7** shows the composition of the complexes before and after reconstitution with CyanoP. The active dimer retained only trace amounts of CyanoP whereas binding to the monomeric assembly intermediate is stoichiometric. These results strongly support our previous conclusion that the extrinsic subunits and especially PsbO block the binding site of CyanoP.

## DISCUSSION

Based on the results of the SPR strategy developed in this study CyanoP was precisely localized at the same position as PsbO in the PSII crystal structure (Umena et al., 2011). In addition, selective binding of CyanoP to a PSII assembly intermediate was shown by in vitro reconstitution experiments. These findings do not only shed light on the structure of CyanoP-PSII complexes but also on the function of this formerly enigmatic protein: as PsbO was shown to be specific for the main subfraction of oxygen-evolving cyanobacterial PSII complexes (Nowaczyk et al., 2006), CyanoP can only be part of transient PSII species related to assembly and/or repair – a hypothesis that was discussed already (Thornton et al., 2004; Nagarajan and Burnap, 2012; Bricker et al., 2013). Although the effects of CyanoP-deletion in *Synechocystis* sp. PCC 6803 are limited (Thornton et al., 2004; Ishikawa et al., 2005; Summerfield et al., 2005; Sveshnikov et al., 2007) there are some indications for this proposed function: PPL1, the closest homolog of CyanoP in the PsbP superfamily of green algae and higher plants (Sato, 2010), was shown to be required for efficient PSII assembly and repair under high light stress (Ishihara et al., 2007). Unfortunately, this effect was neither reported nor excluded for any of the CyanoP deletion mutants, but notably Eaton-Rye and coworkers (Summerfield et al., 2005) noticed reduced growth under elevated temperature, a condition known to cause inactivation of PSII (Allakhverdiev et al., 2007). In concordance, a variation of charge separation properties, which was noticed in a comparative study of the different CyanoP deletion mutants (Sveshnikov et al., 2007), might also be attributed to improperly assembled PSII reaction centers (RCs) as discussed recently (Bricker et al., 2013).

Our results – in particular the interaction with CP43 – support a role of CyanoP in the late phase of PSII biogenesis as binding of CP43 is believed to be the latest step in the assembly of the membrane intrinsic part (Komenda et al., 2012). Also, the association of CyanoP with the thylakoid membrane (Ishikawa et al., 2005) argues for it having a role in late PSII biogenesis as in the early phase PSII was shown to be localized in the plasma membrane (Zak et al., 2001) or in specific thylakoid centers (Stengel et al., 2012). CyanoP may be involved in metal delivery (Nagarajan and Burnap, 2012), which is supported by the presence of zinc ions in its crystal structure (Michoux et al., 2010) and reduced growth of the deletion mutant in media devoid of calcium (Thornton et al., 2004; Summerfield et al., 2005).

**FIGURE 8:**
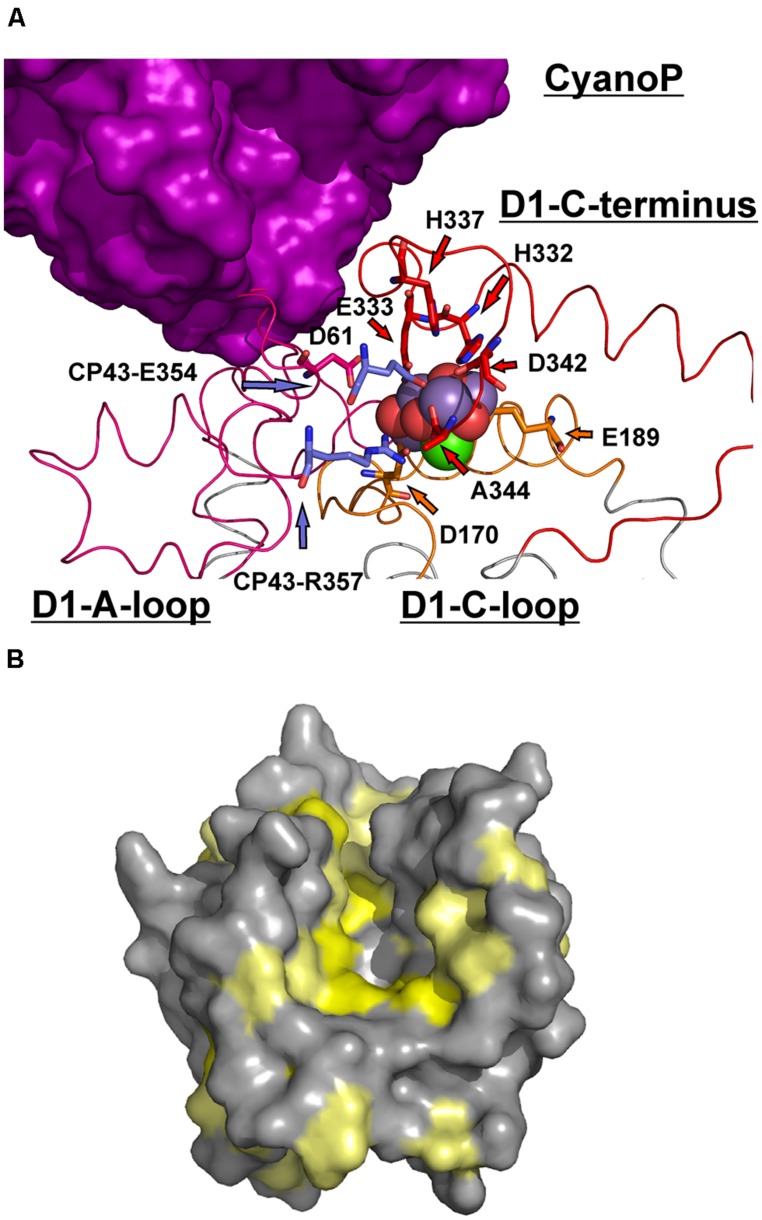
**Implications of the interaction between CyanoP and the D1 C-terminus. (A)** Comparison of the suggested position of CyanoP with the coordination environment of the OEC. D1 is shown in ribbon presentation (a-loop: pink, c-loop: orange, C-terminus: red, transmembrane helices: gray). The ligands including the two residues of CP43 are depicted as sticks, whereas the atoms of the cluster are shown as spheres (oxygen: red, manganese: purple, calcium green). The 13 C-terminal residues of D1 are wound around the OEC and bury the c-loop. If this region adopts a more outstretched conformation in the absence of the OEC, a broad interaction interface with CyanoP is possible. Moreover, the c-loop can be accessed by free metal ions required for the assembly of the OEC. **(B)** Analysis of conserved residues on the surface of CyanoP (Michoux et al., 2010; pdb code: 2XB3) based on a previous study (Jackson et al., 2012). The Consurf-Server (Landau et al., 2005) identified a highly conserved groove on the surface of CyanoP representing an ideal binding site for the D1 C-terminus. Residues with the first and second highest degree of conservation are shown in yellow, the third highest degree in pale yellow and all other residues in gray.

Interestingly, the last 13 amino acids of the mD1 C-terminus (D1pep) alone – including H332, E333, H337, D342, and A344 that are involved in the coordination of the Mn_4_O_5_Ca cluster – are sufficient for a measureable interaction with CyanoP. According to our binding model this highly conserved part of D1 is in close proximity to a groove build by conserved residues (Jackson et al., 2012) on the surface of CyanoP (**Figure 8**). Together with the result that CyanoP binds to a PSII assembly intermediate, which is free of manganese (Mamedov et al., 2007), we came to the hypothesis that the free D1 C-terminus might be coordinated by CyanoP during PSII assembly and might assist the incorporation of manganese. This idea is supported by different experimental findings: (a) the assembly and photoactivation of the Mn_4_O_5_Ca cluster is accompanied by structural changes due to oxidation of the first manganese atom and the concomitant binding of calcium (Chen et al., 1995; Becker et al., 2011); (b) the D1 c-loop is solvent exposed during assembly of the cluster, as D170 and E189 must be accessible for binding of free manganese and calcium (Nixon and Diner, 1992). As the carboxyl group of the C-terminal D1 alanine residue (A344) is one of the calcium ligands it is reasonable that the D1 C-terminus is part of the calcium induced structural change; (c) the fast assembly of the cluster after incorporation and oxidation of the second manganese atom is based on the fact that the protein ligands are already in their final conformation (Tamura and Cheniae, 1987). The role of CyanoP in this process might be to assist the incorporation of calcium by binding to the D1 C-terminus in order to keep the calcium-binding site accessible. The binding of calcium and the simultaneous structural change of the D1 C-terminus could trigger the affinity of CyanoP that would explain its release from the PSII complex during assembly.

Additionally, CyanoP might facilitate the incorporation of CP47 and CP43 into the PSII complex. Taking into account that the RC complex is built by D1 and D2 in the early phase of PSII biogenesis (Komenda et al., 2012), and that these subunits provide the domains with the highest affinity for CyanoP, its binding to the RC complex seems likely. The additional interactions of CyanoP with CP47 and CP43 might facilitate their sequential association to the RC complex to form RC47 (comprising D1, D2, and CP47) and subsequently the monomeric PSII-Psb27 complex (comprising D1, D2, CP47, CP43, and Psb27 as additional extrinsic assembly factor). However, this suggestion requires also further investigation

In this study we have shown that our experimental setup – small-scale expression of solvent-exposed fragments or domains of membrane intrinsic subunits, direct purification on sensor surfaces and SPR interaction analysis with their soluble binding partners – allows precise identification of the protein factor binding sites. As this approach is solely based on recombinant proteins and thereby overcomes problems caused by complex instability, low abundance or transient nature, it is expected that this methodology will be used to analyze the interaction network of other transmembrane protein complexes with their soluble binding partners.

## Conflict of Interest Statement

The authors declare that the research was conducted in the absence of any commercial or financial relationships that could be construed as a potential conflict of interest.
