# Supplementary material for: Localization of the CyanoP binding site on photosystem II by surface plasmon resonance spectroscopy
Source: Front Plant Sci. 2014 Nov 5;5:595. doi: 10.3389/fpls.2014.00595 (PMC4220643; doi:10.3389/fpls.2014.00595)
Supplement: Supplementary file 1 [file Data_Sheet_1.PDF]

# *Supplementary Material*

# Localization of the CyanoP binding site on photosystem II by surface plasmon resonance spectroscopy

**Kai U. Cormann[1], Maik Bartsch[1], Matthias Rögner[1], Marc M. Nowaczyk[1]***

[1] Plant Biochemistry, Ruhr University Bochum, Bochum,

*** Correspondence:** Marc M. Nowaczyk, Plant Biochemistry, Ruhr University Bochum, Universitätsstr. 150, 44801 Bochum, Germany.
Marc.M.Nowaczyk@rub.de

## 1. Supplementary Data

### 1.1. Molecular cloning

Oligonucleotides used for PCR are summarized in table S1. Each fragment was amplified from genomic DNA of *Thermosynechococcus elongatus* with the exception of Im7 and the sequences of the mature and the processed C-terminus of PsbA1, which were amplified from plasmids pQE30-DNaseE7/Im7 (Hosse et al., 2009) and PCRscript_A2_KO, respectively (the latter contains the complete *psbA1* gene). Each fragment was subcloned into vector pJET1.2 (Thermo Scientific). For construction of vectors pIVEX2.3IN and pIVEX2.4IN, fragments coding for Im7 and a short linker sequence (GGSG) were cloned into plasmids pIVEX2.3d and pIVEX2.4d (both 5Prime) using *Nco*I/*Nde*I and *Not*I/*Nde*I restriction sites, respectively. A clone of pIVEX2.4IN contained a frame shift within the *Nde*I restriction site introducing a single leucine residue and a stop codon downstream of the linker sequence and was used to obtain his-tagged Im7 without fusion partner. Templates for cell-free protein expression were generated by introducing the gene sequences of the luminal domains of D1a (*tlr1843*), pD1 (*tlr1843*), mD1 (*tlr1843*), D2 (*tlr1630*), CP43 (*tlr1631*), CP47 (*tlr1530*) and PsbE (*tsr1541*) via *Nde*I/*Xho*I restriction sites into plasmid pIVEX2.3IN.

For generation of plasmid pIVEX2.3IN_D1pep, the coding sequence for Im7 was amplified from plasmid pIVEX2.3IN, and the coding sequence for D1-H332 to D1-A344 (*tlr1843*) was added using an overhang of the reverse primer. The resulting PCR fragment was subcloned into plasmid pJET1.2 and cloned into pIVEX2.3d using *Nco*I and *Xho*I restriction sites.

Plasmids used for *in vivo* expression of Im7-tagged fusion proteins were obtained by *Nde*I/*Xho*I restriction of the pJET1.2 plasmid containing the subcloned fragments of CyanoP (*tlr2075*), CyanoQ (*tll2057*), PsbO (*tll0444*), PsbU (*tll2409*) and Psb27 (*tll2464*) and subsequent ligation into plasmid pIVEX2.4IN. The same fragments of CyanoP and PsbO were also cloned into pIVEX2.4d to obtain an expression vector for the his-tagged form of the proteins. PsbV (*tll1285*) was introduced into plasmid pASK-IBA4 (IBA) via restriction with *Eco*31I.

### 1.2. Heterologous protein overexpression and purification

For *in vivo* expression of Im7 and Im7 fusion proteins of CyanoP, CyanoQ, PsbO, PsbU and Psb27, *E. coli* Overexpress C43 cells (Lucigen) were transformed with the corresponding expression vector

derived from pIVEX2.4IN. For expression of Im7-tagged proteins and Im7 without fusion partner, 500 µl of an overnight grown starter culture were used to inoculate 50 ml of LB-media supplemented with 1 % (w/v) glucose in a 250ml flask. Cultures were continuously grown at 37 °C under vigorous shaking. At an $OD_{600}$ of 0.6 expression was induced by addition of isopropylthiogalactoside to a final concentration of 0.5 mM. After three more hours of growth cells were harvested by centrifugation, resuspended in IMAC equilibration buffer (20 mM MES, 500 mM NaCl, 20 mM imidazole; pH 6.5) and disrupted by sonification. The supernatant was applied on an IMAC gravity flow column and washed with seven column volumes (CV) of IMAC equilibration buffer. The protein was eluted with five CV of IMAC elution buffer (20 mM MES, 500 mM NaCl, 500 mM imidazole; pH 6.5), dialyzed against MBS (20 mM MES, 150 mM NaCl; pH 6.5) and stored at −80 °C.

In principle, expression and cell disruption for his-tagged CyanoP was identical with the exception that four 2l flasks with 500 ml media were each inoculated with 5 ml of starter culture. Purification of CyanoP was performed with an AEKTA Purifier system (GE Healthcare) in two steps using an IMAC and a subsequent anion exchange chromatography. For IMAC the complete supernatant was loaded on a 5ml HisTrap crude FF column (GE Healthcare) and washed with ten CV of IMAC equilibration buffer. The protein was eluted in two steps: first by a linear gradient from 0 to 66 % IMAC elution buffer over four CV and subsequently with 100 % IMAC elution buffer for five CV. Fractions containing predominantly CyanoP were pooled and dialyzed for two times against 1 l IEC equilibration buffer (20 mM MES; pH 6.5). The protein was loaded on a Resource Q 6ml column (GE Healthcare), washed with five CV of IEC equilibration buffer an eluted by a linear gradient from 0 to 1 M NaCl over 20 CV. The eluted protein was dialyzed against MBS and stored at -80 °C. The purification of his-tagged recombinant PsbO was conducted with an identical protocol.

For expression of strep-tagged PsbV, BL21 cells were co-transformed with the PEC86 plasmid (Arslan et al., 1998) and the pASK-IBA4 expression vector containing the mature sequence of PsbV. Eight 2l flasks with 1 l TB media supplemented with 2.5 mM betaine and 500 mM sucrose were each inoculated with 10 ml of starter culture and grown to an $OD_{600}$ of 0.6 at 37 °C under vigorous shaking. After induction with 200 µg/l anhydrotetracycline cultures were slowly stirred at 24 °C for additional 16 h. Cells were harvested, resuspended in TBS (100 mM Tris, 150 mM NaCl; pH 8.0) and disrupted by sonification. The amount of supernatant corresponding to a culture volume of 500 ml was loaded on a Streptactin High Capacity Superflow 1ml column (IBA), washed with twelve CV of TBS and eluted with MBS containing 2.5 mM desthiobiotin. The purified protein was dialyzed against MBS and stored at −80 °C.

### 1.3. SPR experiments

Purification and immobilization of Im7-tagged luminal domains was achieved in a single step using MBS (20 mM MES, 150 mM NaCl, pH 6.5) as running buffer at a flow rate of 30 µl/min and a temperature of 25 °C. For preparation of the reference surface a 1000-fold dilution of the reaction mixture without expression template in immobilization buffer (20 mM MES, 1 M NaCl; pH 6.5) was injected for four minutes. The active surface was prepared similarly using a reaction mixture expressing either the Im7-tagged luminal domain of pD1 (17 kDa), mD1 (16 kDa), D2 (17 kDa), CP43 (25 kDa), CP47 (30 kDa) or PsbE (15 kDa). Immobilization of the purified Im7 fusion proteins of CyanoP (30 kDa), CyanoQ (26 kDa), PsbO (40 kDa), PsbU (24 kDa) and Psb27 (25 kDa) was achieved by injecting a 25 nM solution for 30 s over the active cell. Both surfaces were saturated by injection of 200 nM Im7 for 150 s.

Each interaction analysis with CyanoP or PsbV was performed at 25 °C using MBS as running buffer and a constant flow rate of 30 µl/min. For interaction analysis with PsbO MBS-EP (20 mM MES, 150 mM NaCl, 3 mM EDTA, 0.05 % (v/v) Surfactant P20; pH 6.5) was used as running buffer instead of MBS. After a start-up cycle with running buffer the analyte (CyanoP, PsbO or PsbV) was injected for 1 min, and dissociation was monitored for 3 min using the KINJECT command. At the end of each cycle a 1-min pulse of immobilization buffer was injected over both surfaces to remove residual analyte. CyanoP (21 kDa) was used in a dilution series ranging from 98 nM to 100 µM. PsbV (17 kDa) was injected at concentrations ranging from 98 nM to 50 µM for the Im7-CP43, Im7-mD1, Im7-CP47 and Im7-CyanoP data sets and at concentrations ranging from 125 nM to 64 µM for the Im7-PsbU data set. PsbO (31 kDa) was used at concentrations ranging from 98 nM to 50 µM. Each curve was double-referenced with a blank injection of running buffer (Rich and Myszka, 2001). In case of significant binding each, concentration was run as triplicate, whereas for initial screening of an interaction, each concentration was only measured once. In either case, the concentration series were run in randomized order. At the end of each experiment immobilized Im7 and Im7-tagged proteins were completely removed from the surface by two consecutive injections of Gentle Elution Buffer (Thermo Scientific) supplemented with 0.05 % Tween20 (1 min) and 10 mM glycine pH 2.0 (30 s) at a flow rate of 60 µl/min. Sensor surfaces were reused several times without significant loss of ligand capacity.

Steady state responses were averaged from 20 to 5 s before the end of each injection and fitted to a one-site binding isotherm using implemented functions of BIAevaluation Software v 4.1 (GE Healthcare). Precisely, the steady state affinity equation for a one-site binding isotherm is ($R_{eq}$, steady state response; $K_A$, association equilibrium constant; C, analyte concentration; $R_{max}$, maximal surface binding capacity; $K_D$, dissociation equilibrium constant):

$$R_{eq} = \frac{K_A \cdot C \cdot R_{max}}{K_A \cdot C + 1} \quad \text{with: } K_A = \frac{1}{K_D}$$

## 2. Supplementary Figures and Tables

### 2.1. Supplementary Tables

**Supplementary Table 1. Oligonucleotides used for PCR**

| Sequence | Amplificat |
|---|---|
| ATTCATATGTCGGCCACCAGTGGG | CyanoP |
| TTCTCGAGTTAATATACAGTAAACGAGGACAC | CyanoP |
| GTTAATGGTCTCAGCGCCGCCGAACTGACCC | PsbV |
| CTTATTGGTCTCGTATCAGTAATACACTTTGCCACCGCC | PsbV |
| GATATCATATGGGCACCCCACGCC | PsbE |
| GTTGTCTCGAGCTACTTCAACTGTTCTAAGAAGG | PsbE |
| TGACATATGAACCTGAATGGCTTCAACTTC | pD1/mD1 |
| TTCTCGAGTCAGCCGTTGATGCTGG | pD1 |
| ATCTCGAGTCAGGCCAAGTCGAGGG | mD1 |
| CATATGGCTGCTCCCCCTGTGG | D1a |
| CTCGAGCTATTGGTAGGGGCCACCG | D1a |
| CTCGAGTCAGGCCAAGTCGAGGGGGAAGTTGTGAGCATTGCGCTCGTGCATATGGCCTCC GCTGCCGCCCTG | Im7D1pep |
| TCTCATATGCGGTCCTATGACTTCATTTCG | D2 |
| TTCTCGAGTCAGAGAGCGTTACCACGG | D2 |
| TGACATATGAACAACACGGTCTATCCCAG | CP43 |
| ATCTCGAGTCAAGAGCGGGGTGAGAC | CP43 |
| TTACATATGACTCGCTATCAGTGGGATAGTAG | CP47 |
| AACTCGAGTTAACTGGTGCGGAAAATCC | CP47 |
| ATTCATATGGGCGGCCCCAGTGC | CyanoQ |
| AACTCGAGCTAGGACAACTCAGGCAAGC | CyanoQ |
| CATATGGCAAAACAGACTTTAACCTATGAC | PsbO |
| CTCGAGGGCAGGTTCGATGCTGG | PsbO |
| AATCATATGGCAACTGCATCCACCG | PsbU |
| TTCTCGAGCTACTTGTATAGCCCATTATTG | PsbU |
| CATATGGCCAATGTGCCTACGG | Psb27 |
| CTCGAGTTAGGACTTCGCTTCGCG | Psb27 |
| TAGCGGCCGCGAACTGAAAAATAGTATTAGTGATTAC | Im7 |
| CATATGGCCTCCGCTGCCGCCCTGTTTAAATCCTGG | Im7 |
| TTACCATGGAACTGAAAAATAGTATTAGTGATTAC | Im7/Im7D1pep |

**Supplementary Table 2. MS analysis results (see Fig. 7)**

| Band | Identified proteins, top three (SEQUEST protein score*; # unique peptides; # PSMs) |
|---|---|
| 1 | ApcB (218.16; 7; 70), ApcA (141.07; 9; 57), CpcA (134.73; 6; 37) |
| 2 | ApcB (362.08; 10; 111), CpcB (38.48; 5; 12), CpcA (31.6; 5; 9) |
| 3 | Psb28 (30.2; 3; 9), ApcB (18.05; 3; 6), ApcA (6.34, 2; 3) |

* The score is the sum of all peptide Xcorr values above the specified charge dependent threshold.

## 2.2. Supplementary Figures

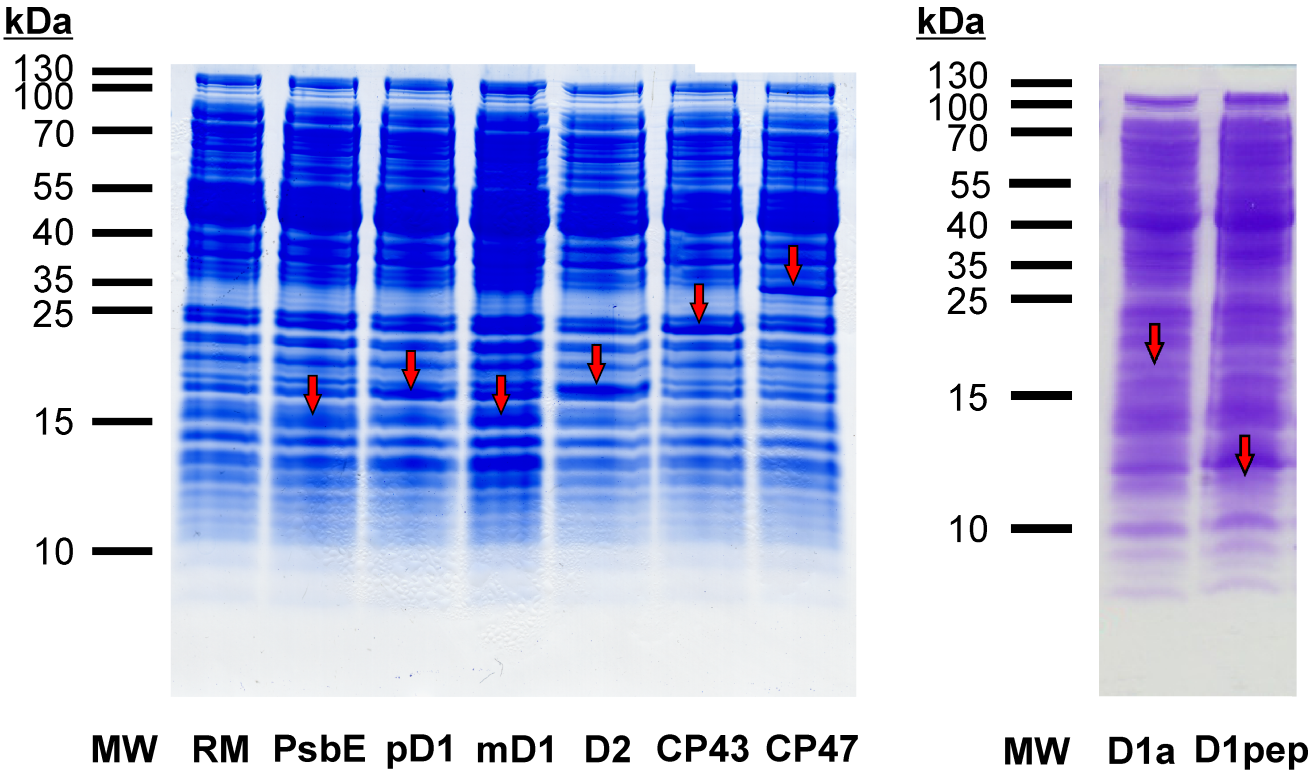


**Supplementary Figure 1. SDS-PAGE of the in vitro expression reactions of the Im7-tagged luminal domains.** Raw reaction mixtures of PsbE (15 kDa), pD1 (17 kDa), mD1 (16 kDa), D2 (17 kDa), CP43 (25 kDa), CP47 (30 kDa) D1a (17 kDa) and D1pep (12 kDa) were loaded on the gel. A comparison of the protein profiles of the corresponding lanes with the reaction mixture without expression template (RM) and the molecular weight standard (MW) reveals additional bands at the expected size (red arrows). The identity of the fusion proteins was confirmed by mass spectrometry (see Fig. S2).

Im7-D1aLoop
MELKNSISDYTEAEFVQLLKEIEKENVAATDDVLDVLLEHFVKITEHPDGTDLIYYPSDNRDDSPEGIVKEIKEWRAANGK
PGFKQGGSGGHMAAPPVDIDGIREPVSGSLLYGNNIITGAVVPSSNAIGLHFYPIWEAASLDEWLYNGGPYQ

Im7-D1pep
MELKNSISDYTEAEFVQLLKEIEKENVAATDDVLDVLLEHFVKITEHPDGTDLIYYPSDNRDDSPEGIVKEIKEWRAANGK
PGFKQGGSGGHMHERNAHNFPLDLA

Im7-mD1
MELKNSISDYTEAEFVQLLKEIEKENVAATDDVLDVLLEHFVKITEHPDGTDLIYYPSDNRDDSPEGIVKEIKEWRAANGK
PGFKQGGSGGHMNLNGFNFNHSVIDAKGNVINTWADIINRANLGMEVMHERNAHNFPLDLA

Im7-pD1
MELKNSISDYTEAEFVQLLKEIEKENVAATDDVLDVLLEHFVKITEHPDGTDLIYYPSDNRDDSPEGIVKEIKEWRAANGK
PGFKQGGSGGHMNLNGFNFNHSVIDAKGNVINTWADIINRANLGMEVMHERNAHNFPLDLASAESAPVAMIAPSING

Im7-D2
MELKNSISDYTEAEFVQLLKEIEKENVAATDDVLDVLLEHFVKITEHPDGTDLIYYPSDNRDDSPEGIVKEIKEWRAANGK
PGFKQGGSGGHMRSYDFISQEIRAAEDPEFETFYTKNLLLNEGIRAWMAPQDQPHENFVFPEEVLPRGNAL

Im7-PsbE
MELKNSISDYTEAEFVQLLKEIEKENVAATDDVLDVLLEHFVKITEHPDGTDLIYYPSDNRDDSPEGIVKEIKEWRAANGK
PGFKQGGSGGHMGTPRPDSYYAQEQRSIPLVTDRFEAKQQVETFLEQLK

Im7-CP43
MELKNSISDYTEAEFVQLLKEIEKENVAATDDVLDVLLEHFVKITEHPDGTDLIYYPSDNRDDSPEGIVKEIKEWRAANGK
PGFKQGGSGGHMNNTVYPSEFYGPTGPEASQAQAMTFLIRDQKLGANVGSAQGPTGLGKYLMRSPTGEIIFGGETMRFWDF
RGPWLEPLRGPNGLDLNKIKNDIQPWQERRAAEYMTHAPLGSLNSVGGVATEINSVNFVSPRS

Im7-CP47
MELKNSISDYTEAEFVQLLKEIEKENVAATDDVLDVLLEHFVKITEHPDGTDLIYYPSDNRDDSPEGIVKEIKEWRAANGK
PGFKQGGSGGHMTRYQWDSSYFQQEINRRVQASLASGATLEEAWSAIPEKLAFYDYIGNNPAKGGLFRTGPMNKGDGIAQA
WKGHAVFRNKEGEELFVRRMPAFFESFPVILTDKNGVVKADIPFRRAESKYSFEQQGVTVSFYGGELNGQTFTDPPTVKSY
ARKAIFGEIFEFDTETLNSDGIFRTS

**Supplementary Figure 2.** Mass spectrometry analysis of IM7 fusion proteins (see Fig. S1). Sequence parts that were identified by MS analysis are highlighted in yellow (IM7: blue; linker: purple; PSII domain: green).

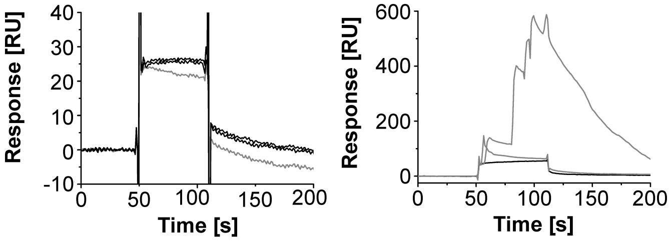


**Supplementary Figure 3. Instrumental errors in the PsbV CP43 and CyanoP D1pep data sets.** Due to baseline drift at the beginning of the injection the SPR signal artificially decreases in one binding curve (left panel; grey line) compared to the constant equilibrium responses shown in Fig. 3 (black lines). Two replicates of 12.5 µM CyanoP (right panel; grey lines) show clear deviations from the well-behaved binding response shown in Fig. 5 (black line).

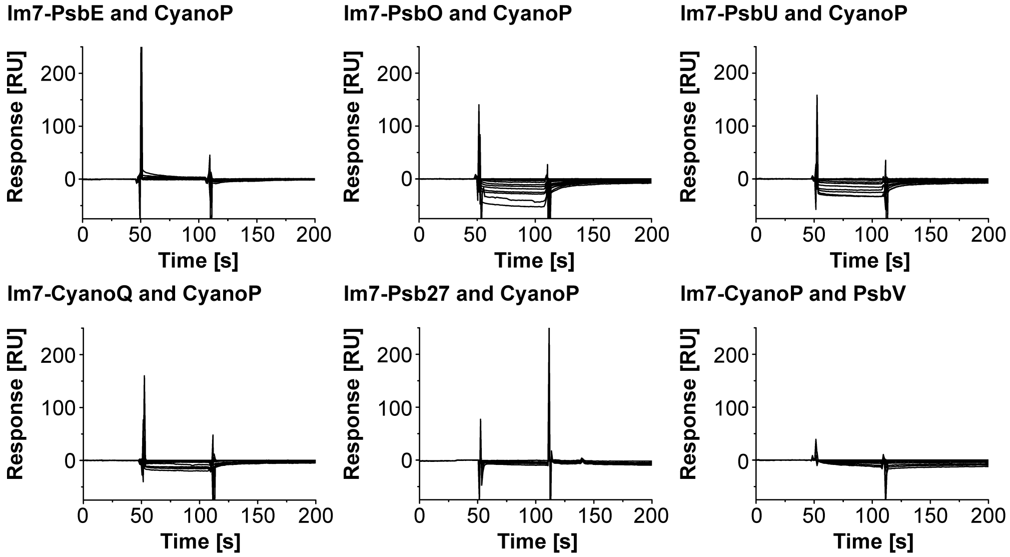


**Supplementary Figure 4. SPR interaction analysis of CyanoP and putative interaction partners (PsbE, PsbO, PsbV, PsbU, CyanoQ and Psb27).** With the exception of the PsbV data set, soluble CyanoP (up to 100 µM) was passed over a surface on which the corresponding Im7 fusion protein was captured. For interaction analysis between CyanoP and PsbV, the latter was injected at concentrations of up to 50 µM over immobilized Im7-CyanoP.

## 3. References

Arslan, E., Schulz, H., Zufferey, R., Kunzler, P., and Thony-Meyer, L. (1998). Overproduction of the Bradyrhizobium japonicum c-type cytochrome subunits of the cbb3 oxidase in Escherichia coli. *Biochem Biophys Res Commun* 251**,** 744-747. doi: 10.1006/bbrc.1998.9549.

Hosse, R.J., Tay, L., Hattarki, M.K., Pontes-Braz, L., Pearce, L.A., Nuttall, S.D., and Dolezal, O. (2009). Kinetic screening of antibody-Im7 conjugates by capture on a colicin E7 DNase domain using optical biosensors. *Anal Biochem* 385**,** 346-357. doi: 10.1016/j.ab.2008.11.026.

Rich, R.L., and Myszka, D.G. (2001). Survey of the year 2000 commercial optical biosensor literature. *J Mol Recognit* 14**,** 273-294. doi: 10.1002/jmr.547.
